# Unraveling the microecological mechanisms of phosphate-solubilizing *Pseudomonas asiatica* JP233 through metagenomics: insights into the roles of rhizosphere microbiota and predatory bacteria

**DOI:** 10.3389/fmicb.2025.1538117

**Published:** 2025-01-28

**Authors:** Yuhan Tang, Linlin Wang, Jing Fu, Fangyuan Zhou, Hailei Wei, Xiaoqing Wu, Susu Fan, Xinjian Zhang

**Affiliations:** ^1^Shandong Provincial Key Laboratory of Applied Microbiology, Ecology Institute, Qilu University of Technology (Shandong Academy of Sciences), Ji’nan, China; ^2^Key Laboratory of Microbial Resources Collection and Preservation, Ministry of Agriculture and Rural Affairs, Institute of Agricultural Resources and Regional Planning, Chinese Academy of Agricultural Sciences, Beijing, China

**Keywords:** phosphate-solubilizing bacteria, *Pseudomonas asiatica*, soil P cycling, metagenomics, predatory bacteria

## Abstract

The effects of phosphate-solubilizing bacteria (PSB) on plant productivity are high variable under field conditions. Soil phosphorus (P) levels are proposed to impact PSB performance. Furthermore, the effect of exogenous PSB on rhizosphere microbial community and their functions are largely unexplored. Our study examined how different P background and fertilization affected the performance of PSB *Pseudomonas asiatica* JP233. We further conducted metagenomic sequencing to assess its impact on rhizosphere microbiota and functions, with a focus on genes related to soil P cycling. We found that JP233 could enhance P solubilization and tomato growth to different extent in both high and low P soils, irrespective of P fertilization. It was particularly effective in high P soil without extra fertilization. JP233 altered the rhizosphere microbial community, boosting taxa known for plant growth promotion. It also changed soil gene profiling, enriching pathways related to secondary metabolite biosynthesis, amino acids, carbon metabolism, and other key processes. Particularly, JP233 increased the abundance of most P cycle genes and strengthened their interconnections. Populations of certain predatory bacteria increased after JP233 inoculation. Our findings provide valuable insights into PSB’s mechanisms for P solubilization and plant growth promotion, as well as potential adverse impacts of resident microbes on bioinoculants.

## 1 Introduction

Macroelements in soil participate in various physiological activities of crop growth and development, and are closely related to crop yield (Wang F. et al., 2022; Wang L. L. et al., 2022; Wang Z. H. et al., 2022; Pan and Cai, 2023; Zhang et al., 2023). After nitrogen, phosphorus (P) is the second nutritional element that limits terrestrial ecosystems the most (Yang and Post, 2011). The amount of P that is currently directly absorbed and utilized by plants is less than 2.5% of the total P concentration in the world’s soil, which severely limits plant growth and development (Pan and Cai, 2023; Pang et al., 2024). Nevertheless, conventional P application techniques not only fail to address this issue, but also lead to critical environmental consequences, such as eutrophication (Zhang et al., 2017; Feng et al., 2019). Phosphate-solubilizing bacteria (PSB) can liberate soluble P from recalcitrant P sources in the soil, providing plants with directly utilizable P. This microbial inoculant has been widely studied as an eco-friendly strategy to promote P uptake by plants (Luo et al., 1993; Feng et al., 2019; Huang K. et al., 2024; Huang Y. L. et al., 2024).

PSB, recognized as plant growth promoting rhizobacteria (PGPR), can be inoculated into the plant rhizosphere to enhance soil available phosphorus (AP) content and promote plant growth, and there have been many successful cases using PSB as P bio-fertilizers to enhance plant productivity (Bargaz et al., 2021; Elhaissoufi et al., 2022). However, there are also many studies report that effects of PSB in improving plant growth is variable, particularly under field conditions and their performances are influenced by many environmental factors (Raymond et al., 2021). For example, the soil total P and AP were found to have relations with PSB populations (Li et al., 2021). Furthermore, colonization and persistence of PSB in soil was proposed to be as a prerequisite for expression of P-solubilization and PGPR traits (Raymond et al., 2021).

A vast array of microbes resides within the rhizosphere, where their interactions with plants are crucial for nutrient acquisition and growth enhancement (Trivedi et al., 2020). Recently, considerable efforts have been directed toward understanding whether exogenous bioinoculants can successfully colonize in the rhizosphere and the subsequent effects they may have on the indigenous microbial community (Da Silva et al., 2022; Swiatczak et al., 2023; Sierra-García et al., 2024). Many studies have revealed that, although bioinoculants do not dominate the soil microbiome, they can indirectly foster plant growth by modulating the structure of the rhizosphere microbiome (Liu et al., 2020; Hu et al., 2021). However, due to the bioinoculants’ intricate roles in the rhizosphere, their impacts on the resident microbiome remain largely unexplored (Swiatczak et al., 2023). Specifically, most research has centered on the impact of inoculants on the structure of the resident microbiota, with no studies yet reporting on the effects of exogenous PSB on microbiome functions (Kong and Liu, 2022). PSB are pivotal in soil P cycling, which are mainly regulated by four gene groups related to P-starvation response regulation, P-uptake and transport, inorganic P-solubilization, and organic P-mineralization (Liu et al., 2023). As of now, the influence of exogenous PSB on the P cycle gene profiles of resident microbiome remains unknown.

*Pseudomonas asiatica* JP233 is an efficient PSB isolated in our laboratory. Previous studies have demonstrated its ability to solubilize P and promote plant growth, with 2-keto gluconic acid (2KGA) serving as the primary functional factor for P solubilization *in vitro* (Yu et al., 2022). The *gcd* gene, which is crucial for the production of 2KGA, has been identified in its genome (Wang F. et al., 2022; Wang L. L. et al., 2022; Wang Z. H. et al., 2022). To devise optimal application strategies for JP233 in agricultural settings, the primary objectives of this study are as follows: Firstly, to investigate how varying P levels in soil affect the P solubilization and growth-promotion capabilities of JP233. Secondly, to utilize metagenomics sequencing to elucidate the impact of exogenous PSB on the structure and functional genes of plant rhizosphere microbiomes, with special emphasis on the effects on genes associated with soil P cycling.

## 2 Materials and methods

### 2.1 Plant and bacterial

*P. asiatica* JP233, isolated from the soil of vegetable greenhouse in Shouguang, Shandong Province, has been proved to be a potentially efficient Phosphate-solubilizing bacteria (PSB) (Wang F. et al., 2022; Wang L. L. et al., 2022; Wang Z. H. et al., 2022; Yu et al., 2022). Bacterial strains were streaked and purified on Luria-Bertani (LB) solid medium, single colonies were selected and transferred to LB liquid medium for shake culture at 180 rpm and 28°C for 24 h. The bacterial culture was then centrifuged, with the supernatants being discarded, and washed and re-suspended with sterile water as inoculant. Two kinds of soil with different phosphorus (P) (Supplementary Tables S1, S2), high-P soil and low-P soil, were used in the plant pot experiment, and Dutch hard powder tomato varieties were purchased from local suppliers.

### 2.2 Effect of different phosphorus levels on the solubility of phosphorus

The experiment involved 12 treatments, utilizing two different P levels soils [low-P (L) and high-P (H)]. Three levels of P application at 0, 50, and 100 mg kg^−1^ were applied under varying P levels soils. The experimental group was inoculated with the JP233 strain, while the control group received an equal amount of water. Each treatment included 12 biological replicates. The seeds were disinfected with alcohol and sodium hypochlorite, then placed in sterile water-soaked petri dishes at 28°C to aid germination. Subsequently, the tomato seedlings were transplanted into planting cups. When the tomato reached true leaf stage, the experimental group was inoculated with JP233 bacterial solution by root irrigation, and each plant received a 6 mL bacterial suspension with 10^8^ CFU mL^−1^. The control group received the same amount of sterile water. The pot experiment was run in a controlled environment at 25°C in full light in the greenhouse. Samples were collected at 7, 14, 21, 28 days post-inoculation of the bacterial solution to measure plant height, stem diameter, fresh weight/dry weight of the above/underground plant parts, total P content in the plant parts, total phosphorus (TP) content in the soil, and available phosphorus (AP) content in the soil. Plant total P was analyzed using the H_2_SO_4_-H_2_O_2_ digestion method and vanadium molybdate blue colorimetric method (Bao, 2000), soil TP was determined using the potassium persulfate digestion method (Su and Chen, 2010), and soil AP was assessed using the Olsen method (Bao, 2000).

### 2.3 DNA extraction and metagenomic sequencing

The H0 (0 mg kg^−1^ P added in high P soil) level, which is the most significant level of increase in soil AP content, was selected and the tomato rhizosphere soil was sampled for metagenomic sequencing analysis. The rhizosphere soil was collected by taking tomato root from its pot and gently shaking off the loosely attached bulk soil. The soil firmly adhering to the roots, with a thickness of approximately 1 mm, was deemed as rhizosphere soil and gathered through a washing and centrifugation process. DNA from rhizosphere soil samples of H0 tomato was isolated and purified with magnetic bead genomic DNA extraction kit (Beijing, Bioteke, China). Using the truSeq nano DNA LT library preparation kit (Illumina, United States), a DNA library was created. LC Bio Technology CO., Ltd. (Hangzhou, China) used the Illumina NovaseqTM 6000 platform to sequence DNA libraries. PE150 was the sequencing mode used. Using the fastqc program,^1^ quality control of the original data was carried out. To eliminate host contamination, reads were aligned to the host genome using bowtie2.^2^ To ensure that the subsequent assembly and analysis results were microbial sequences (Langmead and Salzberg, 2012). Valid data of each sample assembled by MEGAHIT software^3^ to acquire FASTA format file (Li et al., 2015). Coding Region (CDS) prediction was carried out using MetaGeneMark software to retain contigs sequences with a length greater than 500 bp. Sequences with CDS length less than 100 nt were filtered according to the predicted results (Zhu et al., 2010; Gurevich et al., 2013). To extract non-redundant unigenes, CD-HIT^4^ was used to cluster the CDS sequences of all samples. In the meantime, the longest sequence was chosen as the representative sequence to build the Unigenes collection, and identity 95% and coverage 90% were employed for clustering (Fu et al., 2012). The different annotation information taxonomic levels of species were obtained by using DIAMOND software^5^ to compare the Unigenes protein sequence and Non-Redundant Protein Sequence Database (NR) meta library. Compared with Kyoto Encyclopedia of Genes and Genomes (KEGG), Gene Ontology (GO), Evolutionary genealogy of genes: Non-supervised Orthologous Groups (eggNOG), functional databases to obtain annotation information of each functional database (Buchfink et al., 2015). Finally, the abundance informations of various species and functional taxonomic levels were obtained based on the abundance statistics of Unigenes. Statistical analysis was performed based on species, function and Unigenes among the comparison groups. The difference threshold was *p* < 0.05 and |log_2_(fold change)| ≥ 1 (log_2_FC), which was applied for subsequent analysis.

### 2.4 Functional annotation and species classification of soil P cycle genes

Based on the genes related to P cycle in soil microorganisms reported in the literature (Dai et al., 2020; Liang et al., 2020; Huang K. et al., 2024; Huang Y. L. et al., 2024; Wang et al., 2024), a total of 33 genes involved in P cycle were collected by comparing the gene name and functional classification with KEGG annotation information of metagenomic clustering Unigene set, excluding genes involved in intracellular phosphatase production in microbial metabolic activities. According to the function of genes, they can be divided into three categories: (1) Genes involved in P-starvation response regulation; (2) Genes involved in P-uptake and transport system; (3) Genes involved in inorganic P-solubilization; (4) Genes involved in organic P-mineralization. The names, functions and classifications of genes related to P cycle in soil microorganisms are shown in (Supplementary Table S3). Based on the corresponding relationship between species and function in the samples, the correlation between species abundance and functional abundance was analyzed. DIAMOND software was used to compare Unigenes protein sequence with NR database (blastp, evalue≤1e−5). Each Unigene with the best comparison result index was selected as the species classification (Buchfink et al., 2015), and Unigene, species (all hierarchical species) and function (all hierarchical annotation results) were annotated accordingly.

### 2.5 Statistical analysis

Student’s *t* test (*T*-test) and homogeneity of Variance Test were performed using IBM SPSS 25.0 (IBM Corporation, Armonk, NY, United States) to examine the significant differences between different P treatments. Metagenomic data using OmicStudio tools for bioinformatics analysis.^6^ The functional abundance tables of all Unigenes and eight functional databases were statistically analyzed by Mann Whitney U test, and the threshold value of |log_2_FC| ≥ 1, (*p* < 0.05) was used to determine the significant differences. The four types of genes involved in P-cycling were mapped with Unigenes and visualized in the volcano map. The results of KEGG Pathway Definition functional nonparametric difference analysis were visualized in STAMP plot. The gene abundance of four major types of microorganisms involved in P-cycling, as identified in metagenomic data sets, was normalized using Z-score transformation in R (Wu et al., 2022). The differences were then evaluated using a two-sample *T*-test, and GraphPad Prism 10.0 (GraphPad Software, La Jolla, CA, United States) was used to show the results. Spearman was used for correlation calculation, and the correlation threshold was |rho| > 0.5 and *p* < 0.05. The correlation network map was used to compare the complexity of gene association between the two groups.

## 3 Results

### 3.1 Effect of different P levels on JP233 efficacy

**Figure 1:**
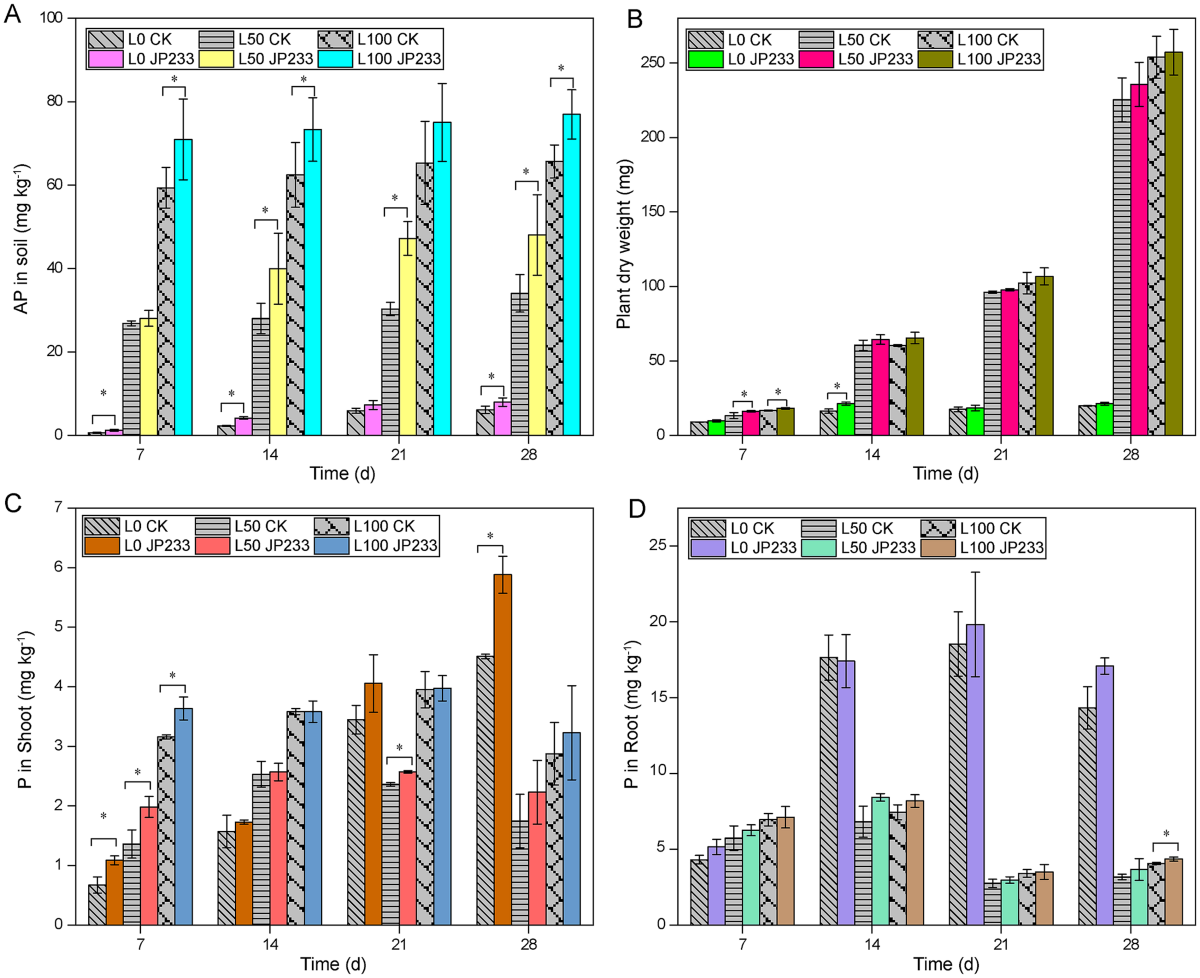
Available phosphorus (AP) content in low phosphorus (P) soil **(A)**; Aboveground dry weight in low-P **(B)**; Content of total phosphorus (TP) in aboveground **(C)**/ underground **(D)** of plants in low-P soil; Results represent means ± Standard Deviation (SD). Homogeneity of Variance Test and Student’s *t* test (*T*-test) analysis were performed for each P level in the experimental group and the control group for the same culture time. * Significant difference was present at *p* < 0.05. L0, L50, L100 (low-P soil supplemented with 0, 50, 100 mg kg^−1^ of KH_2_PO_4_).

To investigate the impacts of P status on PSB, strain JP233 was inoculated to tomato plants cultivated in two kinds of soil with low (L) and high (H) P background. Different P fertilization levels were also included for both soils by amending 0, 50, 100 mg kg^−1^ of KH_2_PO_4_ (designated as L0, L50, L100, H0, H50, H100). In low P soil, JP233 could enhance soil AP within 28 days post inoculation (dpi) across all three P fertilization levels (Figure 1A). Generally, the growth of tomato was much better under L50 and L100 compared to L0. Although JP233 could also promote plant growth, the increase of aboveground dry weight was not statistically significant after 14 dpi (Figure 1B; Supplementary Table S4). Strain JP233 could increase the P content of aboveground plant, and the increment was significant within 7 dpi (Figure 1C). Similarly, it could also increase the P content of tomato root, albeit the increment was not significant (Figure 1D). Notably, the root P content under L0 was much higher than the roots under L50 and L100 after 14 dpi, potentially due to the stressed growth conditions of the tomato plants under L0 (Figures 1B,D).

**Figure 2:**
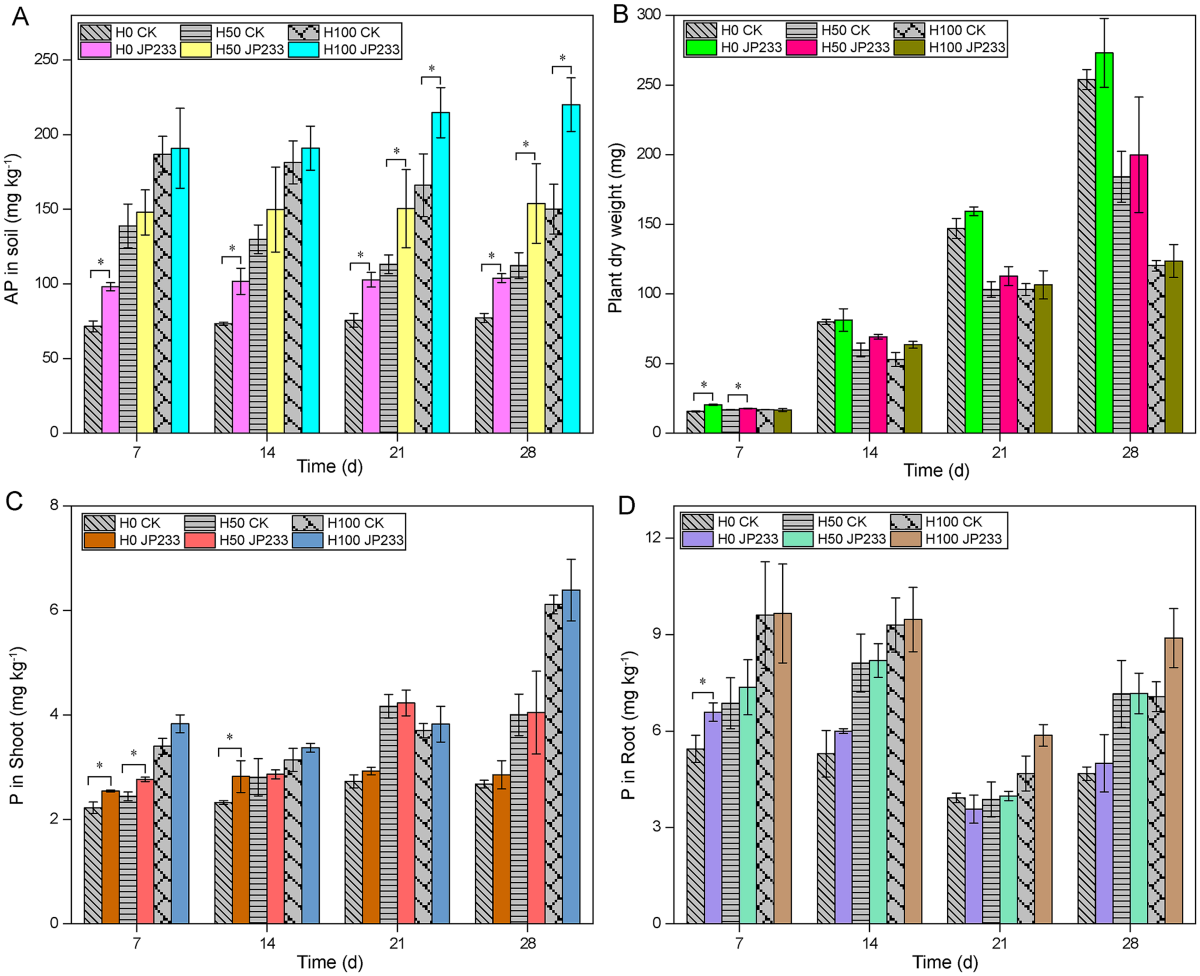
Available phosphorus (AP) content in high phosphorus (P) soil **(A)**; Aboveground dry weight in high-P **(B)**; Content of total phosphorus (TP) in aboveground **(C)**/ underground **(D)** of plants in high-P soil; Results represent means ± SD. Homogeneity of Variance Test and *T*-test analysis was performed for each P level in the experimental group and the control group for the same culture time. * Significant difference was present at *p* < 0.05. H0, H50, H100 (high-P soil supplemented with 0, 50, 100 mg kg^−1^ of KH_2_PO_4_).

In high P soil (Supplementary Table S4), strain JP233 could also enhance soil AP during 28 dpi across all three P fertilization levels (Figure 2A). It is worth noting that extra P fertilization was not beneficial for tomato plant growth, as showed by the reduced aboveground dry weight under H50 and H100 compared with H0 (Figure 2B; Supplementary Table S5). Similar to in low P soil, JP233 could also promote plant growth and enhance the P content of aboveground plant parts and root, albeit the increment was not significant after 7 dpi (Figures 2B–D; Supplementary Table S5). After a comprehensive comparison, the H0 treatment was selected for further metagenomics analysis, as JP233 demonstrated effective P solubilization and plant growth promotion, and the influence of exogenous P could also be minimized under H0 conditions.

### 3.2 Effect of JP233 inoculation on microbial community structure

**Figure 3:**
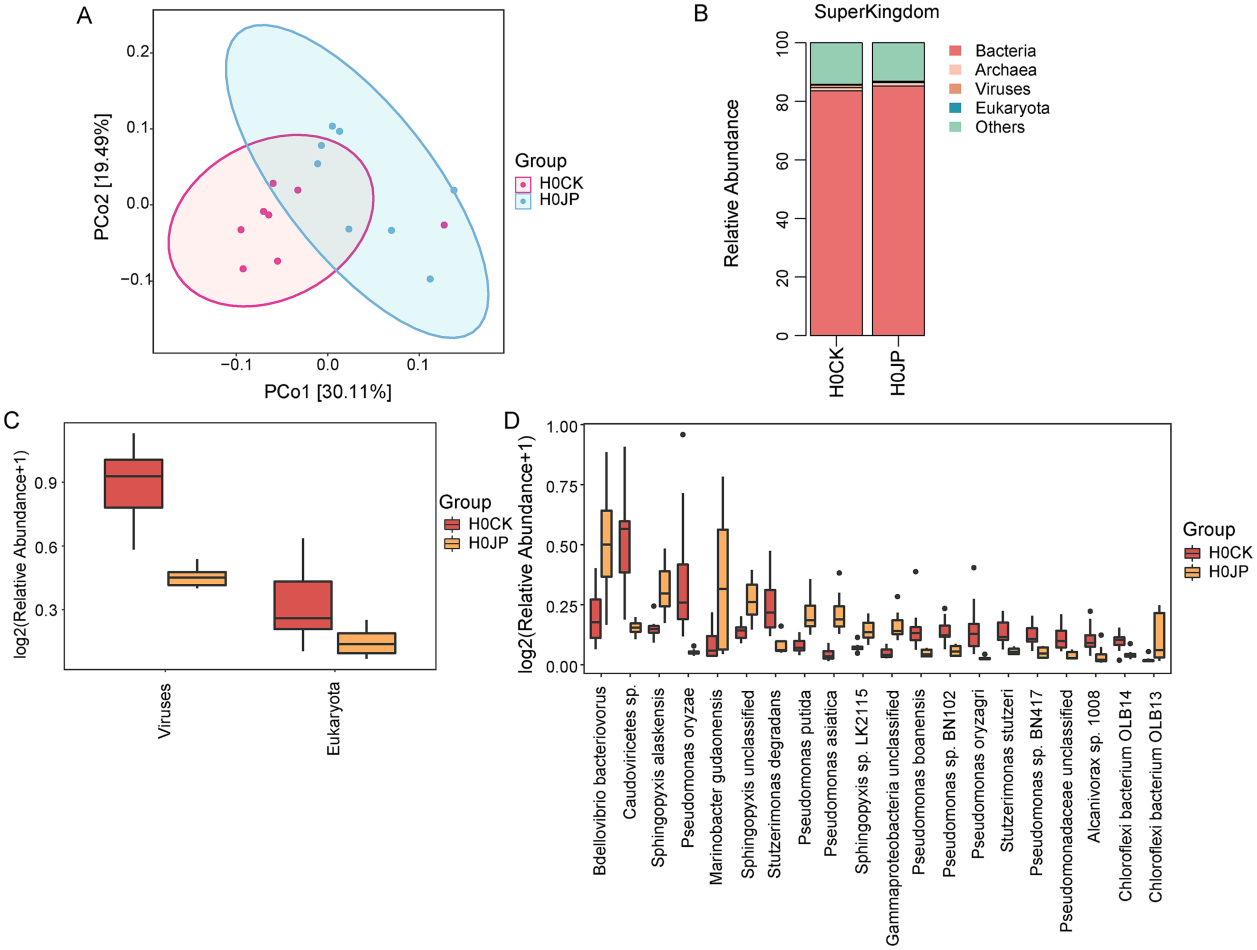
Principal Coordinate Analysis (PCoA) of tomato rhizosphere bacterial community **(A)**; Comparison of differences between two groups of JP (JP233) and CK at super-kingdom level **(B)**; Changes in relative abundance of eukaryotes and viruses with significant differences at super-kingdom level **(C)**; Relative abundance of differentiated species across treatments in both JP and CK groups at the species level **(D)**. Threshold screening is based on |log_2_ Fold Change| ≥ 1 (log_2_FC), *p* < 0.05.

As indicated by Chao1, Shannon and Simpson indices, JP233 inoculation did not significantly change the α-diversity of microbial community (Supplementary Figure S1). However, the Anosim analysis revealed that the JP233 inoculation significantly altered the β-diversity (*p* < 0.05). The Principal Coordinate Analysis (PCoA) showed that JP233-treated soil samples clustered separately from the control (CK), with PCo1 explained 30.11% variance (Figure 3A). Species annotation based on Unigene sequence assembly discovered that the majority of Unigenes in tomato rhizosphere soil (>83%) originated from bacteria. Following JP233 inoculation, the relative abundance of bacteria was enhanced, while the abundance of viruses and eukaryotes were both significantly depressed (*p* < 0.05) (Figures 3B,C). The top 20 abundant species that were altered by JP233 are shown in Figure 3D. As expected, the relative abundance of *P. asiatica* was found enhanced significantly (*p* < 0.05). *Pseudomonas putida* was also found to be elevated, but there is reason to speculate that the reads may have originated from JP233, given the very close phylogenetic relationship between *P. putida* and *P. asiatica* (Wang F. et al., 2022; Wang L. L. et al., 2022; Wang Z. H. et al., 2022). It is noteworthy that the relative abundance of *Bdellovibrio bacteriovorus* significantly increased after JP233 inoculation (Figure 3D). Given that *B. bacteriovorus* is a predatory bacterium that invades and preys on other Gram-negative bacteria (Strauch et al., 2007), the increase has a detrimental effect on the survival of JP233.

### 3.3 Effect of JP233 on rhizosphere soil gene profiling

**Figure 4:**
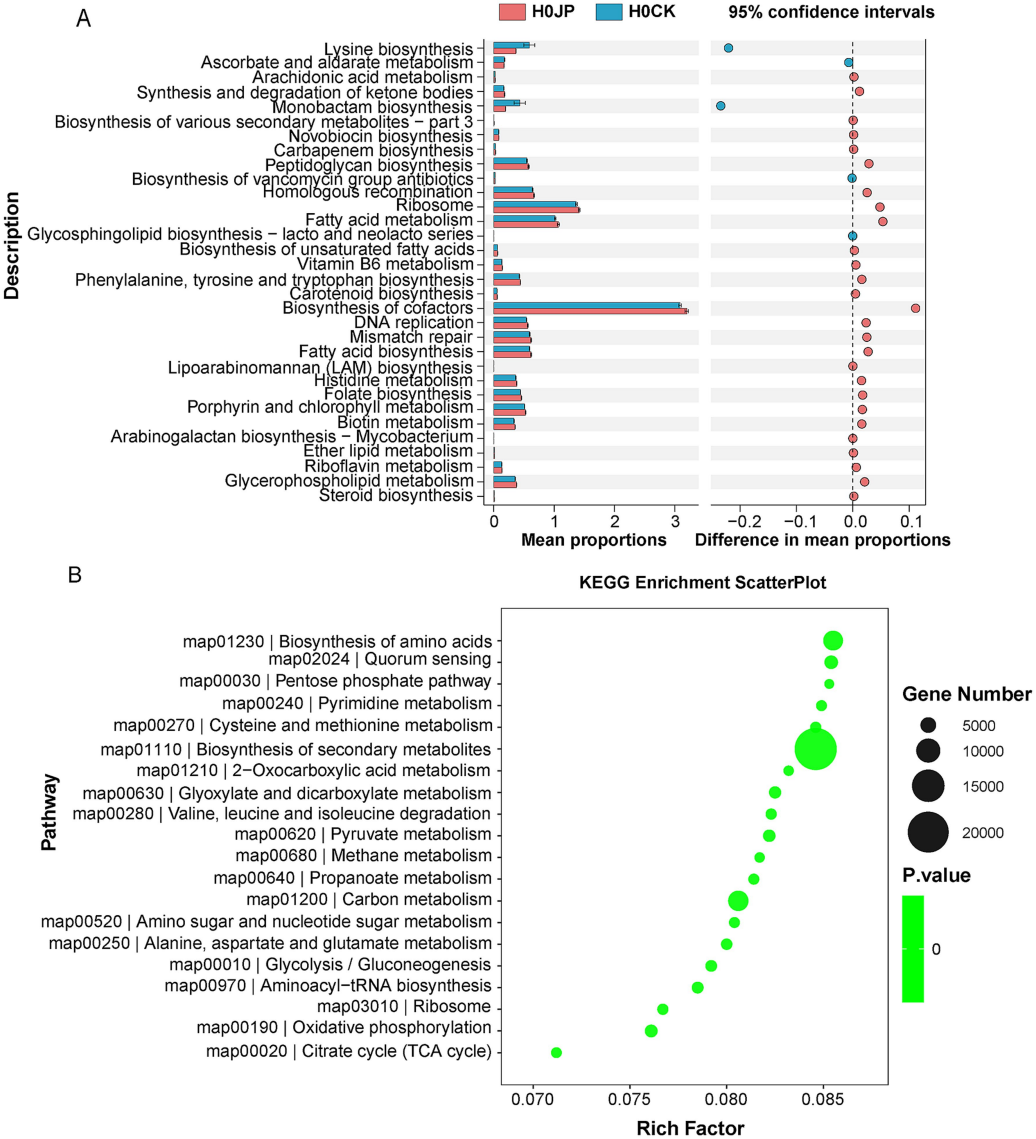
Differences in gene and pathway expression in rhizosphere soil inoculated with JP233 (JP). Kyoto Encyclopedia of Genes and Genomes (KEGG) Pathway Definition entries with significant differences **(A)**; KEGG enrichment analysis was performed for genes with different abundance **(B)**. There were eight samples replicates in each group, and the threshold was |log_2_ Fold Change| ≥ 1 (log_2_FC), *p* < 0.05.

The inoculation of JP233 induced modifications in the rhizosphere soil gene profiling, resulting in a total of 134,184 unigenes with increased abundance and 164,550 unigenes with decreased abundance (Supplementary Figure S2). According to KEGG annotation, these unigenes with significant abundance changes can be categorized into 33 pathways (Figure 4A). Among them, biosynthesis of cofactors, fatty acid metabolism, and ribosome, among others, were enhanced, whereas pathways such as monobactam biosynthesis, lysine biosynthesis, ascorbate and aldarate metabolism exhibited decreases. Enrichment analysis revealed that the unigenes exhibiting significant abundance alterations were predominantly enriched in pathways including the biosynthesis of secondary metabolites, amino acids, carbon metabolism, and others (Figure 4B). Given the advantageous roles of secondary metabolites, amino acids, and organic carbon in fostering plant health and growth, these alterations in gene profiling could potentially be attributed to the enhancement of plant growth.

### 3.4 Effect of JP233 on genes related to soil P-cycling

**Figure 5:**
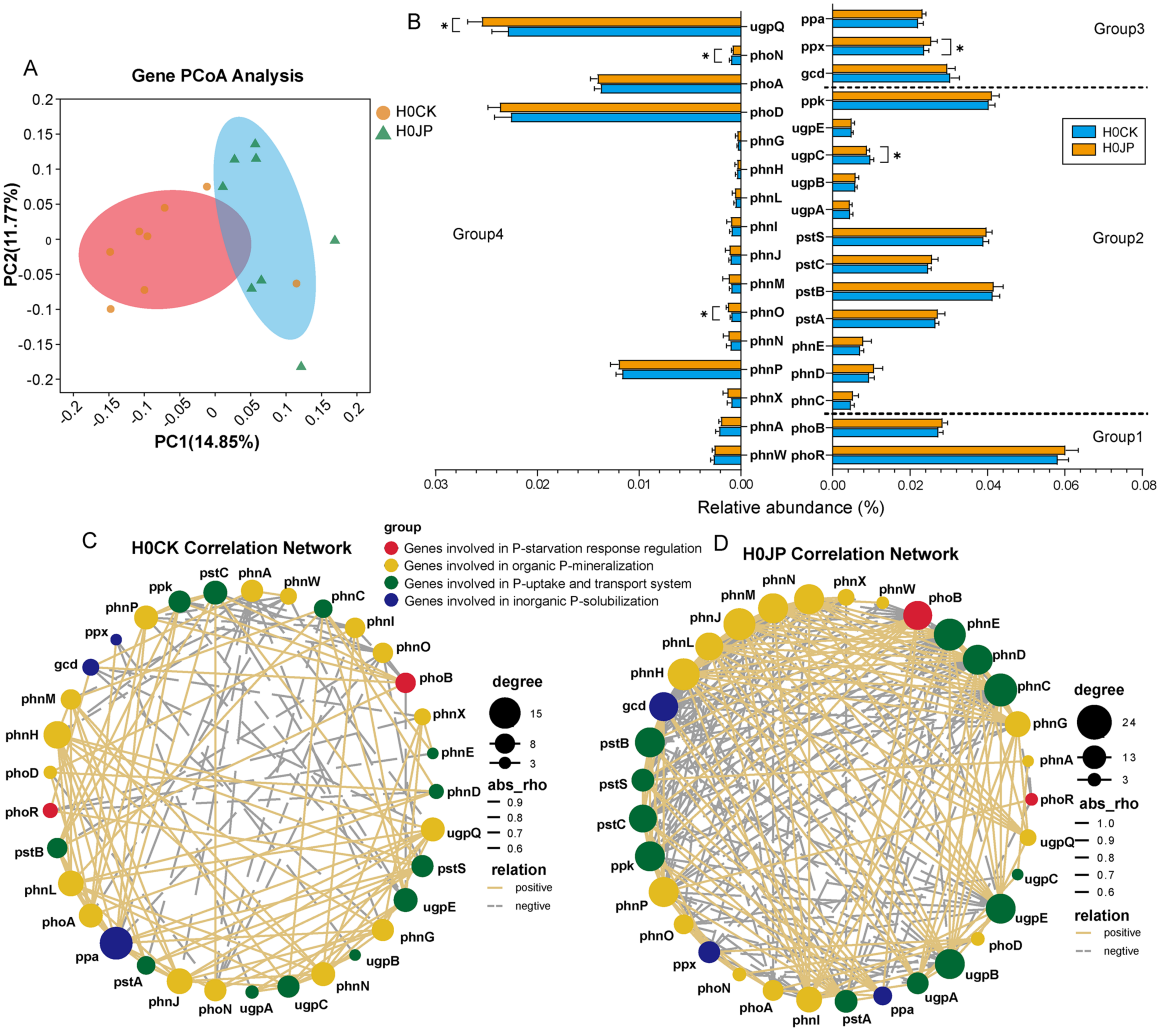
PCoA analysis of P cycle genes **(A)**; Changes in abundance and interaction complexity of genes associated with P cycle gene under JP and CK treatments. Under different treatments, relative abundance of genes involved in (1) P-starvation response regulation, (2) P-uptake and transport system, (3) inorganic P-solubilization, (4) organic P-mineralization **(B)**; Network co-occurrence of genes involved in P cycle between the two groups **(C,D)**. * Indicates a significant difference in the relative abundance of genes between the two groups (*p* < 0.05). Node size and color shade indicate the number of related objects between genes. The thickness of the lines indicates the strength of the correlation between genes. The shape of the lines indicates positive and negative correlations between genes.

The processes of the soil P cycle are primarily governed by four distinct gene groups, which are associated with the regulation of P-starvation response, P-uptake and transport, inorganic P-solubilization, and organic P-mineralization (Liu et al., 2023). Based on KEGG annotation, our focus was on 33 genes involved in P cycle (Supplementary Table S3), excluding genes related to intracellular phosphatase production during microbial metabolism (Bergkemper et al., 2016). Notably, the inoculation of JP233 resulted in a significant 3.4% increase in the total relative abundance of P cycle genes (*p* < 0.05). Furthermore, approximately 79% of these P cycle genes exhibited a heightened abundance. According to PCoA analysis, there were significant difference in the profiling of soil P cycle genes attributable to the inoculation of JP233 (Figure 5A).

The most prominent P cycle genes identified in the soil samples were *phoR*, *pstB*, *ppk*, *pstS* and *gcd* (Figure 5B). Specifically, *phoR*, which encodes the phosphate regulon sensor histidine kinase, is associated with the P-starvation response. The *pstB* and *pstS* genes encode components of the high-affinity phosphate-specific transporter *PstSABC*, while the *ppk* gene, which encodes polyphosphate kinase, is also involved in P-uptake and transport. Additionally, the *gcd* gene, encoding quinoprotein glucose dehydrogenase, is linked to inorganic P-solubilization through gluconic acid production. Despite JP233 possessing the *gcd* gene, it did not significantly increase its abundance in the soil (*p* > 0.05). However, JP233 did enhance the abundance of the *ppx* gene, which encodes exopolyphosphatase (PPX), playing a crucial role in the degradation of inorganic polyphosphate into phosphate (Song et al., 2020). Additionally, JP233 increased the abundances of two genes related to organic P-mineralization: *ugpQ* and *phnO*, which encode glycerophosphodiester phosphodiesterase (GDPD) and a C-P lyase subunit, respectively (*p* < 0.05). Conversely, JP233 also decreased the abundances of two P cycle genes, *ugpC* and *phoN*, which encode components of the Ugp transport system and C-P lyase, respectively (*p* < 0.05).

In addition to the individual P cycle genes, the inoculation of JP233 also modified their co-occurrence network. Specifically, JP233 boosted the connectivity among soil P cycle genes. Comparing with the JP233-treated soil samples, the P gene network in control (CK) soil exhibited lower complexity and sparser linkage density (Figure 5C). The inoculation of JP233 significantly improved key topological parameters, including the average degree and the interconnections among P cycle genes. Notably, genes implicated in P-uptake and transport (*phnCDE*, *pstB*), inorganic P-solubilization and organic P-mineralization (*gcd*, *phnJLHMNP*), as well as the regulation of P-starvation response (*phoB*) demonstrated a substantial increase in node degree and interaction patterns (Figure 5D).

### 3.5 Effect of JP233 on the taxonomic compositions of P cycle genes

**Figure 6:**
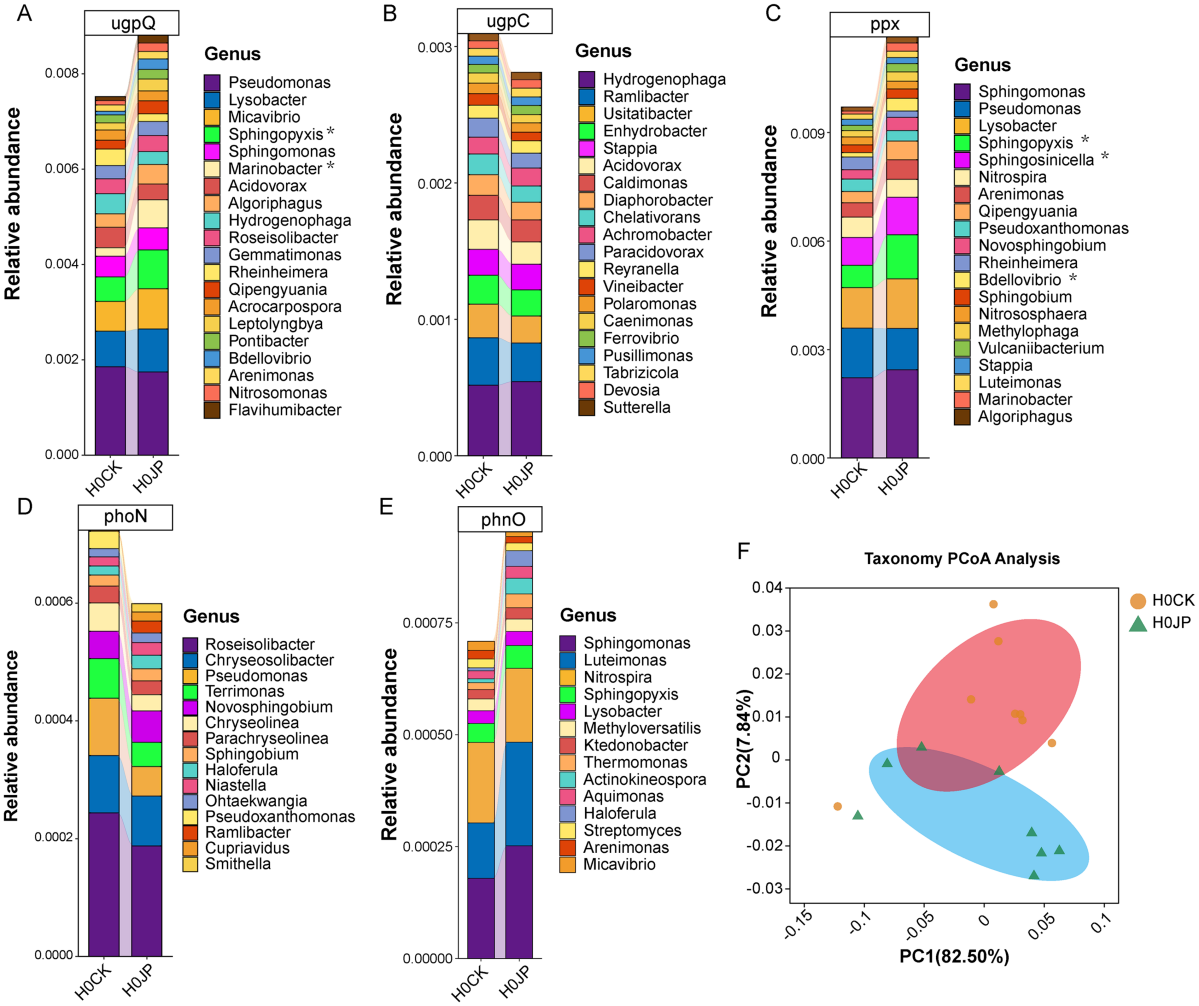
Changes in species corresponding to genes associated with rhizosphere soil P cycle. After the inoculation of JP233, the top 20 genera with the highest content in the significantly altered P cycle genes *ugpQ* **(A)**, *ugpC* **(B)**, *ppx* **(C)**, *phoN* **(D)**, *phnO* **(E)**. PCoA analysis based on Bray-Curtis distance **(F)**.

To ascertain the origins of the alterations in the abundance and co-occurrence network of soil P cycle genes following the inoculation of JP233, their taxonomic compositions were traced. Figure 6 displays the top 20 most abundant genera of the P cycle genes that underwent significant alterations due to JP233. Notably, the abundances of the *ppx* gene from *Sphingopyxis*, *Sphingosinicella*, and *Bdellovibrio* increased significantly (*p* < 0.05). Similarly, the abundances of the *ugpQ* gene from *Sphingopyxis* and *Marinobacter* also showed a significant increase (*p* < 0.05). However, for the genes *phnO*, *ugpC*, and *phoN*, no individual genus within the top 20 showed significant alterations. While the abundances of other P cycle genes remained largely unchanged, their taxonomic compositions did shift, as evidenced by PCoA analysis (Figure 6F).

## 4 Discussion

In this study, we investigated the influence of various phosphorus (P) conditions and P fertilization on the performance of PSB, specifically *P. asiatica* JP233. Our findings revealed that high P soil without additional P amendment (H0) provides an optimal environment for JP233 to exhibit P solubilization and plant growth promotion. Subsequently, we employed metagenomic sequencing to analyze the effects of JP233 on the microbial community structure and functional gene profiles. Special attention was given to genes associated with soil P cycling, in order to elucidate the impacts of JP233 on these critical processes. To our knowledge, this represents the first report utilizing a metagenomics strategy to study the effects of exogenous PSB on the resident rhizosphere microbiome.

The inoculation of JP233 increased the abundance of most P cycle genes and enhanced the connectivity of the gene co-occurrence network, particularly for the genes related to organic P-mineralization (*phnJLHMNP*), P-uptake and transport system genes (*pstB*, *phnCDE*). To different degrees, JP233 induced an increase in the abundance of genes involved in P-starvation response regulation (*phoB*), inorganic P-solubilization (*ppx*, *ppa*), organic P-mineralization (*ugpQ*), alkaline phosphatase (*phoAD*) and other related genes. In soils with long-term high-P inputs, the richness of soil P cycle genes and their interconnections decreased (Liu et al., 2023). Our result demonstrated that the inoculation of PSB, specifically JP233, could alleviate these detrimental effects. In PSB JP233, the *gcd* gene plays a crucial role in producing 2-keto gluconic acid, which is the key functional agent for P solubilization by JP233 *in vitro* (Yu et al., 2022). Surprisingly, despite the enhancement of *gcd* gene abundance from *Pseudomonas* and changes in its distribution pattern among different genera (Supplementary Figure S3), the inoculation of JP233 did not significantly increase the overall abundance of the *gcd* gene (*p* > 0.05). The *gcd* gene serves as a pivotal biomarker for soil P cycle (Wu et al., 2022), and it is proposed that important ecosystem functions should remain preserved even as the microbial community structure undergoes changes (Allison and Martiny, 2008).

**Figure 7:**
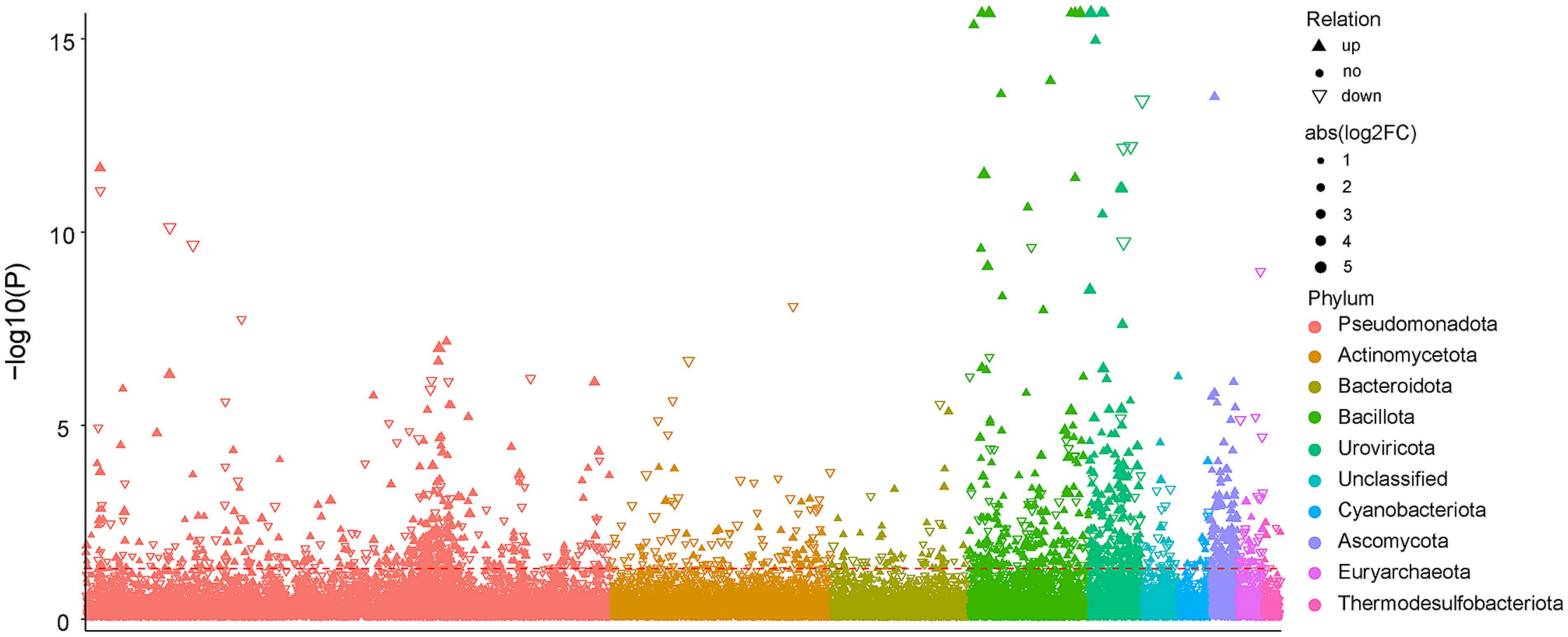
Manhattan plot displays the abundance of enriched bacterial phyla in soil under JP vs. CK treatment at the H0 level. Significantly increased abundance phyla are represented as filled triangles, while significantly decreased abundance phyla are shown as hollow triangles; non-significantly phyla are indicated by circles. The dashed line represents the significance threshold at *p* = 0.05. The color of each point denotes the distinction of the phylum, and the size of each point corresponds to the relative abundance of the phylum.

After inoculating with PSB JP233, significant differences were observed in the microbial community structure of tomato rhizosphere soil (Figure 3A). The influence of exogenous PSB inoculants on the structure of soil microbiota has been widely investigated (Dong et al., 2019; Liu et al., 2020). In this study, the main bacterial phylum in tomato rhizosphere soil, including Pseudomonadota, Acidobacteriota, Bacteroidota, Gemmatimonadota, Chloroflexota, Actinomycetota, Verrucomicrobiota, Myxococcota, Bdellovibrionota, and Planctomycetota, collectively accounted for nearly 81% of the bacterial sequences (Supplementary Figure S4A). Comparable dominant phylum compositions have been reported in other rhizosphere soils (Bejarano et al., 2021; Gao et al., 2023). At the genus level, numerous strains known for their plant-beneficial properties increased following treatment with JP233, including *Sphingomonas* (Wang F. et al., 2022; Wang L. L. et al., 2022; Wang Z. H. et al., 2022), *Lysobacter* (Expósito et al., 2015), *Sphingopyxis* (Dias et al., 2009; Zhang et al., 2019), and *Nitrospira* (Tao et al., 2024) (Figure 4B). In addition to these dominant genera, MetagenomeSeq analysis revealed an enhancement in the relative abundance of various species belonging to *Bacillota* (Figure 7). Notably, among these, species such as *Bacillus* spp. (Probanza et al., 2002), *Paenibacillus* spp. (Li et al., 2021), *Virgibacillus* spp., *Oceanobacillus* spp., *Halobacillus* spp. (Mukhtar et al., 2018), and *Metabacillus* spp. (Yin et al., 2022) have been recognized for their beneficial effects on plants.

**Figure 8:**
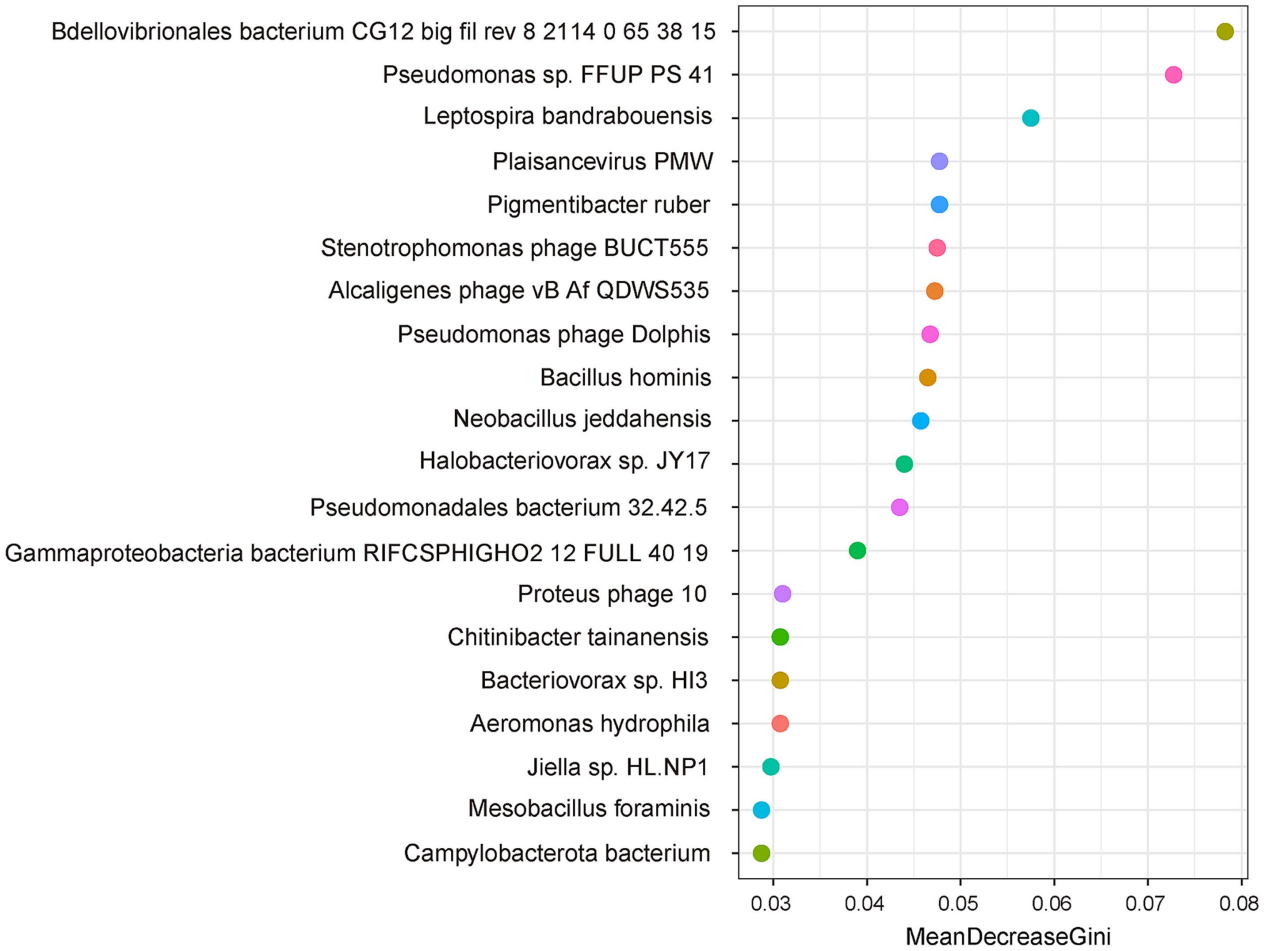
Random Forest analysis revealed the species that play a major role in the classification effect of the JP vs. CK group at the H0 level. The larger the MeanDecreaseGini value, the greater importance of the corresponding variable.

Although the inoculation of JP233 significantly increased the abundance of *P. asiatica*, the overall abundance of *Pseudomonas* declined (Supplementary Figure S4B). This reduction may be partially attributed to the significant surge in *Bdellovibrio bacteriovorus*, a predatory bacterium. At genus level, *Bdellovibrio*, along with *Bacteriovorax*, *Halobacteriovorax* and an unclassified genus from *Bdellovibronales* (Supplementary Figure S5), all exhibited enhanced abundance. Given that many strains from these genera prey on susceptible gram-negative bacteria (Williams et al., 2016; Inoue et al., 2023), their increase could also be a consequence of the JP233 inoculation. The results of random forest analysis indicated that a species from *Bdellovibronales* was the most significant predictor, followed by a species from *Pseudomonas*, for distinguishing the grouping of microbial communities between the non-inoculated control and JP233-treated soil samples (Figure 8). These findings may partially explain the lack of *gcd* gene abundance surge and the weakened effect of JP233 in later stages. Although many studies found the exogenous PGPR could not become the dominant strains, and propose they could indirectly enhance plant growth and health by altering the composition and functionality of the rhizosphere microbial community (Kong and Liu, 2022). Our findings provide deeper insights into the potential adverse impacts of resident microbes on bioinoculants.

## 5 Conclusion

The inoculation of the exogenous PSB strain *P. asiatica* JP233 enhanced P solubilization and promoted tomato growth in soils with both high and low P backgrounds, irrespective of P fertilization levels. Particularly, its impact was potent in high-P soil without additional P fertilization. This inoculation markedly altered the composition of the rhizosphere microbial community, resulting in a decrease in the relative abundances of viruses and eukaryotes. Specifically, the abundances of *Sphingomonas*, *Lysobacter*, *Sphingopyxis*, *Nitrospira*, and certain species from *Bacillota* were elevated. Notably, many strains from these genera and species are known to possess plant growth-promoting traits. Furthermore, the introduction of JP233 altered the soil gene profiling, with the modulated genes predominantly enriched in pathways related to the biosynthesis of secondary metabolites, amino acids, carbon metabolism, and other vital processes. This inoculation also increased the abundance of most P cycle genes and strengthened the interconnections among these genes, thereby contributing to improved P solubilization. The populations of certain predatory bacteria, including those from *Bdellovibrio*, *Bacteriovorax*, and *Halobacteriovorax*, increased after the inoculation of JP233, which may have a detrimental effect on the survival of JP233. The stimulatory impact observed can be attributed to the selective competitive effects of the rhizosphere microbiota, which are directly or indirectly influenced by the inoculated PSB strain JP233. Our findings offer valuable insights into the microecological mechanisms by which PSB solubilize P in soil and promote plant growth.

## Data Availability

The datasets analyzed for this study can be found in the NCBI Sequence Read File (SRA) under project numbers PRJNA1136594.

## Footnotes

^1^http://www.bioinformatics.babraham.ac.uk/projects/fastqc/, V0.10.1.

^2^https://www.sbgrid.org/software/titles/bowtie-2, V2.2.0.

^3^https://github.com/voutcn/megahit, V1.2.9.

^4^http://www.bioinformatics.org/cd-hit/, V4.6.1.

^5^https://github.com/bbuchfink/diamond, V0.9.14.

^6^ https://www.omicstudio.cn/tool
