# Supplementary material for: Unraveling the microecological mechanisms of phosphate-solubilizing Pseudomonas asiatica JP233 through metagenomics: insights into the roles of rhizosphere microbiota and predatory bacteria
Source: Front Microbiol. 2025 Jan 28;16:1538117. doi: 10.3389/fmicb.2025.1538117 (PMC11810911; doi:10.3389/fmicb.2025.1538117)
Supplement: Supplementary file 1 [file Table_1.docx]

Supplementary Tables

**Supplementary Table 1.** Properties of soil with low phosphorus levels

|  | TP (mg kg^-1^) | TN (mg kg^-1^) | AP (mg kg^-1^) | AN (mg kg^-1^) | AK (mg kg^-1^) | pH | OM (%) |
| --- | --- | --- | --- | --- | --- | --- | --- |
| Low fertility soil | 370.75 | 759.56 | 5.01 | 49.25 | 106.62 | 8.78 | 1.51 |

Total phosphorus (TP); Total nitrogen (TN); Available phosphorus (AP); Alkalihydrolyzable nitrogen (AN); Available Potassium (AK); Organic Matter (OM).

**Supplementary Table 2.** Properties of soil with high phosphorus levels

|  | TP (mg kg^-1^) | TN (mg kg^-1^) | AP (mg kg^-1^) | AN (mg kg^-1^) | AK (mg kg^-1^) | pH | OM (%) |
| --- | --- | --- | --- | --- | --- | --- | --- |
| High fertility soil | 2000.24 | 1159.53 | 109.85 | 113.18 | 154.33 | 7.53 | 1.57 |

Total phosphorus (TP); Total nitrogen (TN); Available phosphorus (AP); Alkalihydrolyzable nitrogen (AN); Available Potassium (AK); Organic Matter (OM).

**Supplementary Table 3.** Description and classification of the genes related to soil P cycle

| Classification | KO number | KEGG Gene Description | gene |
| --- | --- | --- | --- |
| Genes involved in P-starvation response regulation | K07636 | two-component system, OmpR family, phosphate regulon sensor histidine kinase | *phoR* |
|  | K07657 | two-component system, OmpR family, phosphate regulon response regulator | *phoB* |
| Genes involved in P-uptake and transport system | K02041 | phosphonate transport system ATP-binding protein | *phnC* |
|  | K02044 | phosphonate transport system substrate-binding protein | *phnD* |
|  | K02042 | phosphonate transport system permease protein | *phnE* |
|  | K02038 | phosphate transport system permease protein | *pstA* |
|  | K02036 | phosphate transport system ATP-binding protein | *pstB* |
|  | K02037 | phosphate transport system permease protein | *pstC* |
|  | K02040 | phosphate transport system substrate-binding protein | *pstS* |
|  | K05814 | sn-glycerol 3-phosphate transport system permease protein | *ugpA* |
|  | K05813 | sn-glycerol 3-phosphate transport system substrate-binding protein | *ugpB* |
|  | K05816 | sn-glycerol 3-phosphate transport system ATP-binding protein | *ugpC* |
|  | K05815  K00937 | sn-glycerol 3-phosphate transport system permease protein  polyphosphate kinase | *ugpE*  *ppk* |
| Genes involved in inorganic P-solubilization | K00117 | quinoprotein glucose dehydrogenase | *gcd* |
|  | K01507 | inorganic pyrophosphatase | *ppa* |
|  | K01524 | exopolyphosphatase / guanosine-5’-triphosphate,3’-diphosphate pyrophosphatase | *ppx* |
| Genes involved in organic P-mineralization | K19670 | phosphonoacetate hydrolase | *phnA* |
|  | K06166 | alpha-D-ribose 1-methylphosphonate 5-triphosphate synthase subunit | *phnG* |
|  | K06165 | alpha-D-ribose 1-methylphosphonate 5-triphosphate synthase subunit | *phnH* |
|  | K06164 | alpha-D-ribose 1-methylphosphonate 5-triphosphate synthase subunit | *phnI* |
|  | K06163 | alpha-D-ribose 1-methylphosphonate 5-phosphate C-P lyase | *phnJ* |
|  | K05780 | alpha-D-ribose 1-methylphosphonate 5-triphosphate synthase subunit | *phnL* |
|  | K06162 | alpha-D-ribose 1-methylphosphonate 5-triphosphate diphosphatase | *phnM* |
|  | K05774 | ribose 1,5-bisphosphokinase | *phnN* |
|  | K09994 | (aminoalkyl) phosphonate N-acetyltransferase | *phnO* |
|  | K06167 | phosphoribosyl 1,2-cyclic phosphate phosphodiesterase | *phnP* |
|  | K03430 | 2-aminoethylphosphonate-pyruvate transaminase | *phnW* |
|  | K05306 | phosphonoacetaldehyde hydrolase | *phnX* |
|  | K01077 | alkaline phosphatase | *phoA* |
|  | K01113 | alkaline phosphatase D | *phoD* |
|  | K09474 | acid phosphatase (class A) | *phoN* |
|  | K01126 | glycerophosphoryl diester phosphodiesterase | *ugpQ* |

**Supplementary Table 4.** Changes of tomato plant after JP233 inoculation in low phosphorus soil

| Phosphorus level | Incubation time (d) | Plant height (cm) | | Stem diameter (mm) | | Aboveground fresh weight (g) | | Underground fresh weight (g) | | Aboveground dry weight (mg) | | Underground dry weight (mg) | |
| --- | --- | --- | --- | --- | --- | --- | --- | --- | --- | --- | --- | --- | --- |
|  |  | CK | JP233 | CK | JP233 | CK | JP233 | CK | JP233 | CK | JP233 | CK | JP233 |
| L0 | 7 | 4.9±0.163a | 5.2±0.125a | 0.8±0.082a | 1.2±0.047b | 0.095±0.002a | 0.119±0.003b | 0.011±0.001a | 0.021±0.001b | 9.0±0.094a | 9.8±0.66a | 1.8±0.125a | 2.1±0.189a |
|  | 14 | 7.8±0.205a | 8.7±0.309b | 1.0±0.047a | 1.3±0.082b | 0.108±0.008a | 0.138±0.01b | 0.017±0.000a | 0.026±0.003b | 16.3±1.476a | 21.4±1.053b | 2.0±0.17a | 2.3±0.294a |
|  | 21 | 8.6±0.287a | 8.8±0.216a | 1.4±0.17a | 1.5±0.287a | 0.142±0.006a | 0.191±0.024a | 0.023±0.002a | 0.027±0.002a | 17.5±1.621a | 18.4±1.893a | 2.1±0.262a | 2.4±0.34a |
|  | 28 | 8.9±0.163a | 9.3±0.047b | 1.5±0.047a | 1.7±0.047b | 0.187±0.001a | 0.213±0.002b | 0.026±0.003a | 0.038±0.005b | 19.9±0.17a | 21.3±0.918a | 3.3±0.17a | 4.3±0.047b |
| L50 | 7 | 5.4±0.082a | 5.6±0.047b | 1.2±0.125a | 1.3±0.141a | 0.110±0.013a | 0.184±0.011b | 0.013±0.002a | 0.022±0.003b | 13.3±2.055a | 16.2±0.535b | 2.2±0.094a | 2.3±0.205a |
|  | 14 | 12.5±0.432a | 13.1±0.685a | 1.3±0.189a | 1.6±0.216a | 0.492±0.014a | 0.516±0.013a | 0.039±0.005a | 0.042±0.004a | 60.5±3.549a | 64.4±3.163a | 6.1±1.034a | 7.0±0.356a |
|  | 21 | 13.9±0.327a | 14.7±0.33a | 1.9±0.309a | 2.0±0.205a | 0.765±0.111a | 1.048±0.104a | 0.074±0.018a | 0.111±0.015a | 96.1±0.785a | 97.8±0.772a | 14.4±0.471a | 21.1±0.852b |
|  | 28 | 15.8±0.33a | 16.6±0.249a | 2.333±0.287a | 2.5±0.125a | 1.241±0.226a | 1.508±0.19a | 0.149±0.006a | 0.183±0.009b | 225.3±46.992a | 235.6±48.155a | 25.0±0.801a | 27.5±2.098a |
| L100 | 7 | 6.0±0.17a | 6.3±0.163a | 1.3±0.125a | 1.4±0.17a | 0.150±0.014a | 0.203±0.011b | 0.021±0.001a | 0.023±0.002a | 16.7±0.368a | 18.2±0.51b | 2.3±0.141a | 2.5±0.082a |
|  | 14 | 12.1±0.432a | 13.2±0.492a | 1.4±0.125a | 1.8±0.356a | 0.424±0.023a | 0.541±0.033b | 0.040±0.001a | 0.043±0.003a | 60.4±0.544a | 65.4±3.872a | 7.6±0.374a | 7.9±0.309a |
|  | 21 | 14.9±0.163a | 15.1±0.591b | 2.2±0.249a | 2.3±0.386a | 1.285±0.024a | 1.403±0.141a | 0.119±0.019a | 0.141±0.012a | 102.3±7.195a | 106.7±5.705a | 18.8±1.996a | 20.1±2.595a |
|  | 28 | 16.2±0.125a | 18.6±0.249b | 2.4±0.17a | 2.5±0.125a | 1.387±0.164a | 1.604±0.193a | 0.179±0.006a | 0.196±0.006a | 253.9±40.949a | 257.3±53.06a | 26.3±0.464a | 27.7±1.066a |

The results are presented as means ± SD. The data were tested for homogeneity of variance test and Student's t test (T-test) and the different lowercase letters in the columns indicate significant differences (*P* < 0.05). L0, L50, L100 (low- phosphorus soil supplemented with 0, 50, 100 mg kg^-1^ of KH_2_PO_4_).

**Supplementary Table 5.** Changes of tomato plant after JP233 inoculation in high phosphorus soil

| Phosphorus level | Incubation time (d) | Plant height (cm) | | Stem diameter (mm) | | Aboveground fresh weight (g) | | Underground fresh weight (g) | | Aboveground dry weight (mg) | | Underground dry weight (mg) | |
| --- | --- | --- | --- | --- | --- | --- | --- | --- | --- | --- | --- | --- | --- |
|  |  | CK | JP233 | CK | JP233 | CK | JP233 | CK | JP233 | CK | JP233 | CK | JP233 |
| H0 | 7 | 6.4±0.094a | 6.9±0.17b | 1.2±0.082a | 1.7±0.17b | 0.182±0.007a | 0.230±0.022b | 0.023±0.002a | 0.037±0.005b | 15.6±0.34a | 20.4±0.464b | 2.1±0.082a | 3.0±0.205b |
|  | 14 | 13.1±0.33a | 16.2±0.294b | 1.5±0.125a | 2.2±0.403a | 0.561±0.031a | 0.764±0.012b | 0.035±0.001a | 0.058±0.003b | 79.8±1.799a | 81.2±8.139a | 9.1±0.634a | 10.8±0.082b |
|  | 21 | 15.4±0.125a | 16.9±0.163b | 2.6±0.082a | 3.0±0.245a | 1.178±0.131a | 1.494±0.026b | 0.080±0.007a | 0.143±0.008b | 147.0±7.118a | 159.3±3.091a | 13.0±0.205a | 17.7±1.601b |
|  | 28 | 15.4±0.249a | 17.9±0.125b | 2.9±0.125a | 3.4±0.17b | 1.530±0.063a | 1.734±0.186a | 0.147±0.01a | 0.222±0.007b | 253.9±7.149a | 273.0±24.78a | 17.0±1.558a | 21.7±3.84a |
| H50 | 7 | 6.4±0.082a | 6.7±0.047b | 1.3±0.125a | 1.6±0.294a | 0.145±0.011a | 0.224±0.011a | 0.022±0.004a | 0.029±0.002a | 16.8±0.245a | 17.7±0.205b | 2.2±0.082a | 2.3±0.141a |
|  | 14 | 1.01±0.294a | 15.0±0.262b | 1.4±0.047a | 2.2±0.613a | 0.368±0.125a | 0.619±0.046b | 0.037±0.005a | 0.051±0.001a | 59.7±4.928a | 69.2±1.676a | 6.1±0.163a | 7.2±0.294b |
|  | 21 | 13.7±0.125a | 16.3±0.216b | 2.1±0.45a | 2.9±0.499a | 1.089±0.129a | 1.358±0.197a | 0.091±0.013a | 0.117±0.009a | 103.1±5.651a | 112.8±6.793a | 14.2±1.731a | 16.6±0.804a |
|  | 28 | 18.0±0.806a | 17.8±0.163a | 2.3±0.294a | 2.4±0.66a | 1.375±0.169a | 1.610±0.279a | 0.115±0.016a | 0.126±0.005a | 184.1±18.258a | 199.8±41.477a | 13.7±0.793a | 16.8±1.236b |
| H100 | 7 | 6.6±0.249a | 6.7±0.17a | 1.4±0.082a | 1.4±0.17a | 0.148±0.006a | 0.133±0.022b | 0.021±0.002a | 0.017±0.004a | 16.8±0.082a | 16.6±1.023a | 1.8±0.386a | 1.9±0.403a |
|  | 14 | 11.8±0.309a | 15.9±0.125a | 1.6±0.125a | 2.4±0.33b | 0.525±0.025a | 0.717±0.037b | 0.039±0.003a | 0.043±0.002a | 52.9±4.965a | 63.5±2.406a | 5.5±0.17a | 6.0±0.125b |
|  | 21 | 13.8±0.249a | 15.8±0.573a | 2.4±0.464a | 2.5±0.356a | 1.187±0.176a | 1.199±0.103a | 0.106±0.012a | 0.095±0.004a | 103.1±4.141a | 106.5±10.14a | 11.5±1.223a | 10.8±0.531a |
|  | 28 | 17.1±0.17a | 17.5±0.309b | 2.4±0.283a | 2.5±0.497a | 1.575±0.074a | 1.641±0.176b | 0.125±0.008a | 0.118±0.011a | 120.2±3.795a | 123.6±11.834a | 14.6±0.66a | 12.8±1.37a |

The results are presented as means ± SD. The data were tested for homogeneity of variance test and T-test and the different lowercase letters in the columns indicate significant differences (*P* < 0.05). H0, H50, H100 (high- phosphorus soil supplemented with 0, 50, 100 mg kg^-1^ of KH_2_PO_4_).
