# Supplementary figures and images for: Unraveling the microecological mechanisms of phosphate-solubilizing Pseudomonas asiatica JP233 through metagenomics: insights into the roles of rhizosphere microbiota and predatory bacteria
Source: Front Microbiol. 2025 Jan 28;16:1538117. doi: 10.3389/fmicb.2025.1538117 (PMC11810911; doi:10.3389/fmicb.2025.1538117)

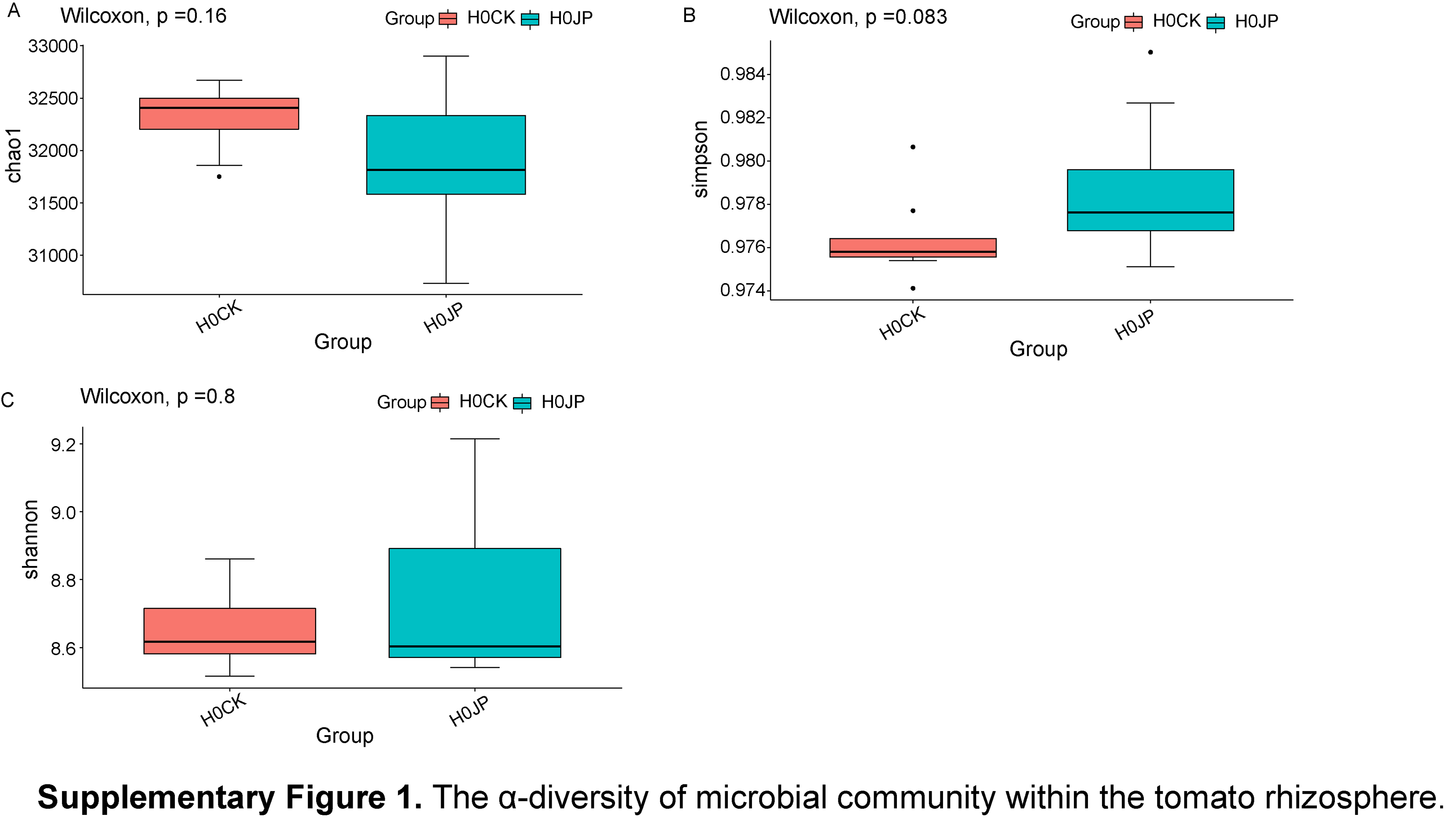

Supplement: Supplementary file 2 [file Image_1.tif]

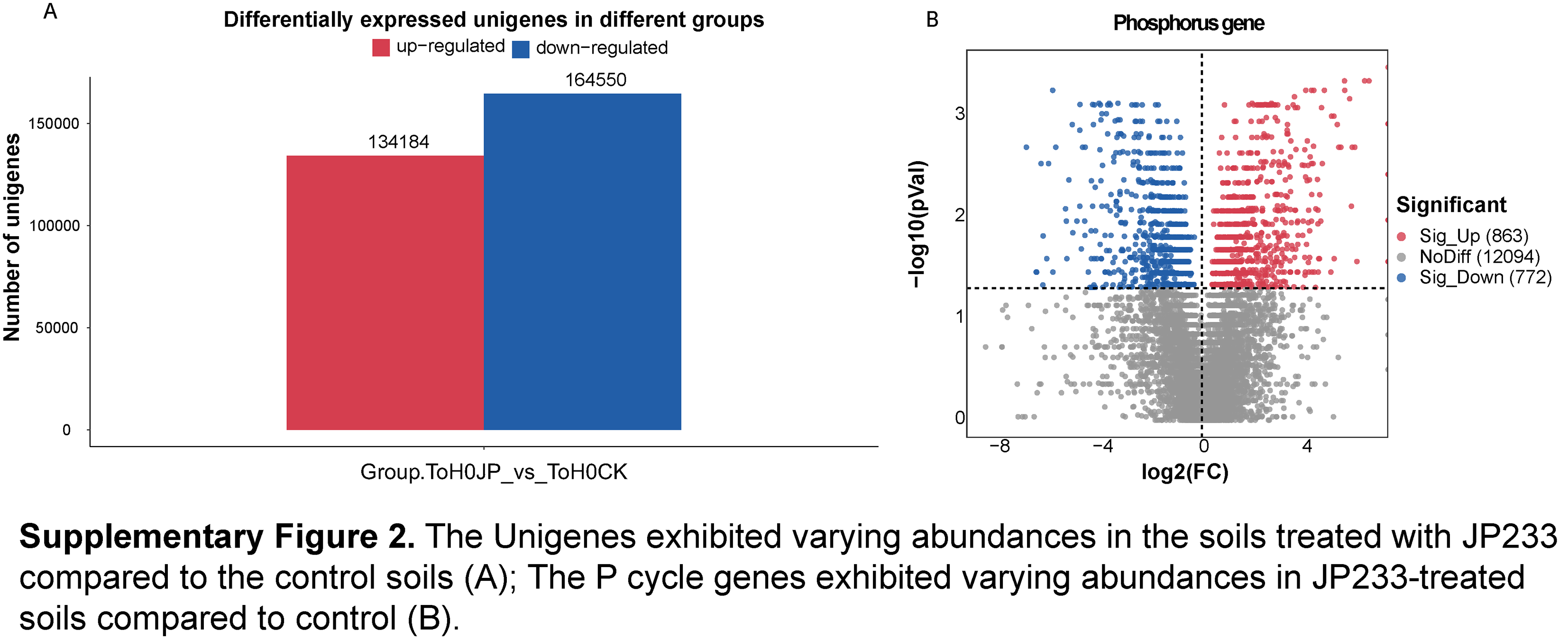

Supplement: Supplementary file 3 [file Image_2.tif]

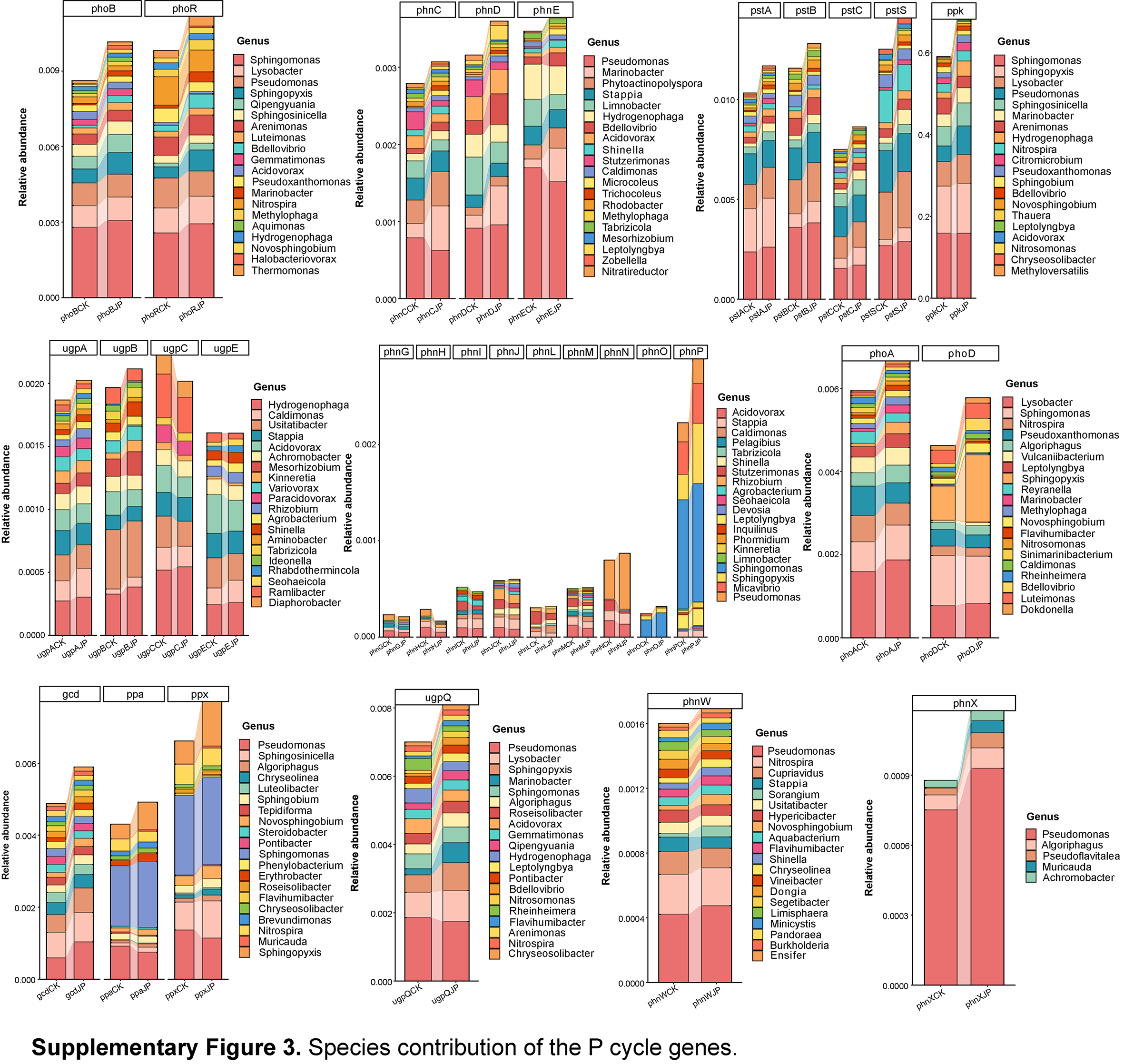

Supplement: Supplementary file 4 [file Image_3.tif]

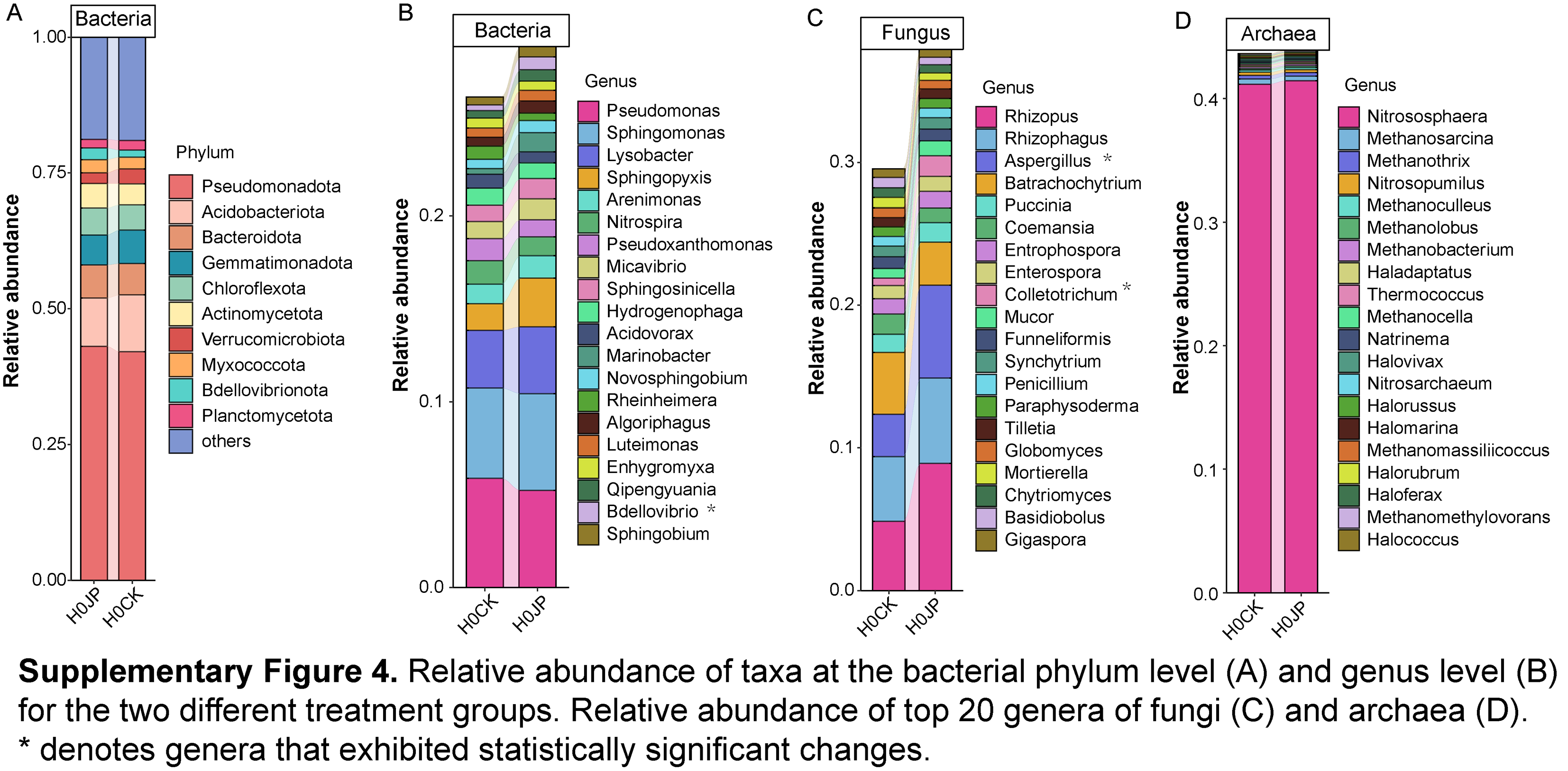

Supplement: Supplementary file 5 [file Image_4.tif]

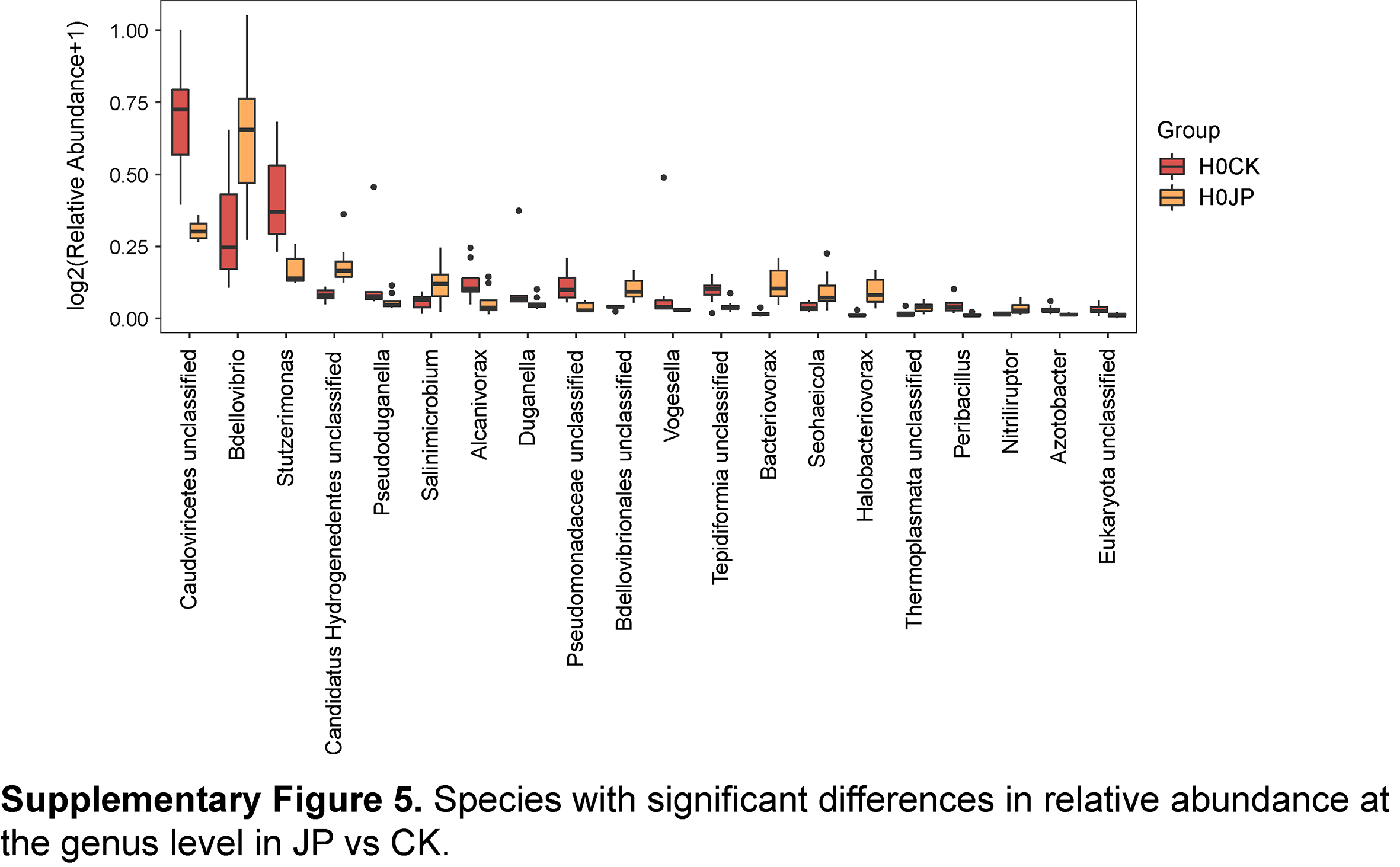

Supplement: Supplementary file 6 [file Image_5.tif]
